# Insights into gene expression profiles induced by *Socs3* depletion in keratinocytes

**DOI:** 10.1038/s41598-017-16155-1

**Published:** 2017-11-20

**Authors:** Archana Bajpai, Takashi Ishii, Kosuke Miyauchi, Vipul Gupta, Yuka Nishio-Masaike, Yuki Shimizu-Yoshida, Masato Kubo, Hiroaki Kitano

**Affiliations:** 1 RIKEN-IMS, Laboratory for Disease Systems Modeling, Yokohama, Japan; 2 RIKEN-IMS, Laboratory for Cytokine Regulation, Yokohama, Japan; 3 The Systems Biology Institute, Tokyo, Japan; 4 Division of Molecular Pathology, Research Institute for Biomedical Science, Tokyo University of Science, Tokyo, Japan; 5 Sony Computer Science Laboratories, Inc, Tokyo, Japan; 6 Okinawa Institute of Science and Technology, Okinawa, Japan

## Abstract

Specific deletion of suppressor of cytokine signaling 3 (*Socs3*) in keratinocytes can cause severe skin inflammation with infiltration of immune cells. The molecular mechanisms and key regulatory pathways involved in these processes remain elusive. To investigate the role of Socs3 in keratinocytes, we generated and analyzed global RNA-Seq profiles from *Socs3* conditional knockout (cKO) mice of two different ages (2 and 10 weeks). Over 400 genes were significantly regulated at both time points. Samples from 2-week-old mice exhibited down-regulation of genes involved in keratin-related functions and up-regulation of genes involved in lipid metabolism. At week 10, multiple chemokine and cytokine genes were up-regulated. Functional annotation revealed that the genes differentially expressed in the 2-week-old mice play roles in keratinization, keratinocyte differentiation, and epidermal cell differentiation. By contrast, differentially expressed genes in the 10-week-old animals are involved in acute immune-related functions. A group of activator protein-1–related genes were highly up-regulated in *Socs3* cKO mice of both ages. This observation was validated using qRT-PCR by SOCS3-depleted human keratinocyte–derived HaCaT cells. Our results suggest that, in addition to participating in immune-mediated pathways, SOCS3 also plays important roles in skin barrier homeostasis.

## Introduction

Skin is an essential organ that consists of two major layers, the epidermis and dermis. The epidermis, the outermost layer of skin tissue, consists of keratinocytes, which perform diverse biological functions. In particular, the epidermis controls multiple intercellular immune responses by producing cytokines that promote maintenance of skin barrier homeostasis^1,2^. Cytokines are essential intercellular mediators of immune signals regulated by SOCS (Suppressor of Cytokine Signaling) family proteins, which communicate extensively with various cellular and molecular responses via the JAK–STAT (Janus kinase–signal transducer and activator of transcription) pathway and dynamically regulate tissue hemostasis^3–5^. Although these systems are robust, perturbation of any of their components can lead to a variety of diseases, including allergy, autoimmune skin diseases, inflammation, and cancers^5–11^.

Recently, several groups extensively investigated the role of SOCS family proteins in skin homeostasis and inflammation, and sought to determine how dysregulation of skin hemostasis leads to altered cutaneous immune responses during inflammatory pathogenesis in the skin^2,12,13^. The role of SOCS3 in immune regulation is well established^14–16^. Moreover, a recent study using transgenic mice showed that dysregulation of *Socs3*, but not *Socs1*, in keratinocytes results in hyper-active immune responses and hyperplasia in the epidermis, eventually leading to a psoriasis-like phenotype^13^. This observation implies that *Socs3* governs cellular processes beyond the immune response; however, the underlying molecular mechanisms and key regulatory pathways underlying the sequential breaching of the skin barrier and induction of inflammation in the absence of *Socs3* remain poorly understood.

To investigate the role of *Socs3* in epidermal homeostasis, we used RNA-Seq to systematically analyze differential gene expression profiles of cells from 2 and 10 week-old *Socs3* cKO mice. We identified the most significantly altered genes, as well as the highly enriched biological pathways and networks crucial for epidermal hemostasis. In addition, we validated our RNA-Seq analysis results by qRT-PCR in cultured human keratinocytes. Our results suggest that *Socs3* not only controls immune homeostasis, but also promotes maintenance of epidermal homeostasis. Our findings provide new molecular insights into how perturbed epidermal homeostasis contributes to the development of skin diseases.

## Results

### Altered gene expression profiles in *Socs3* cKO keratinocytes at 2 and 10 weeks

**Figure 1:**
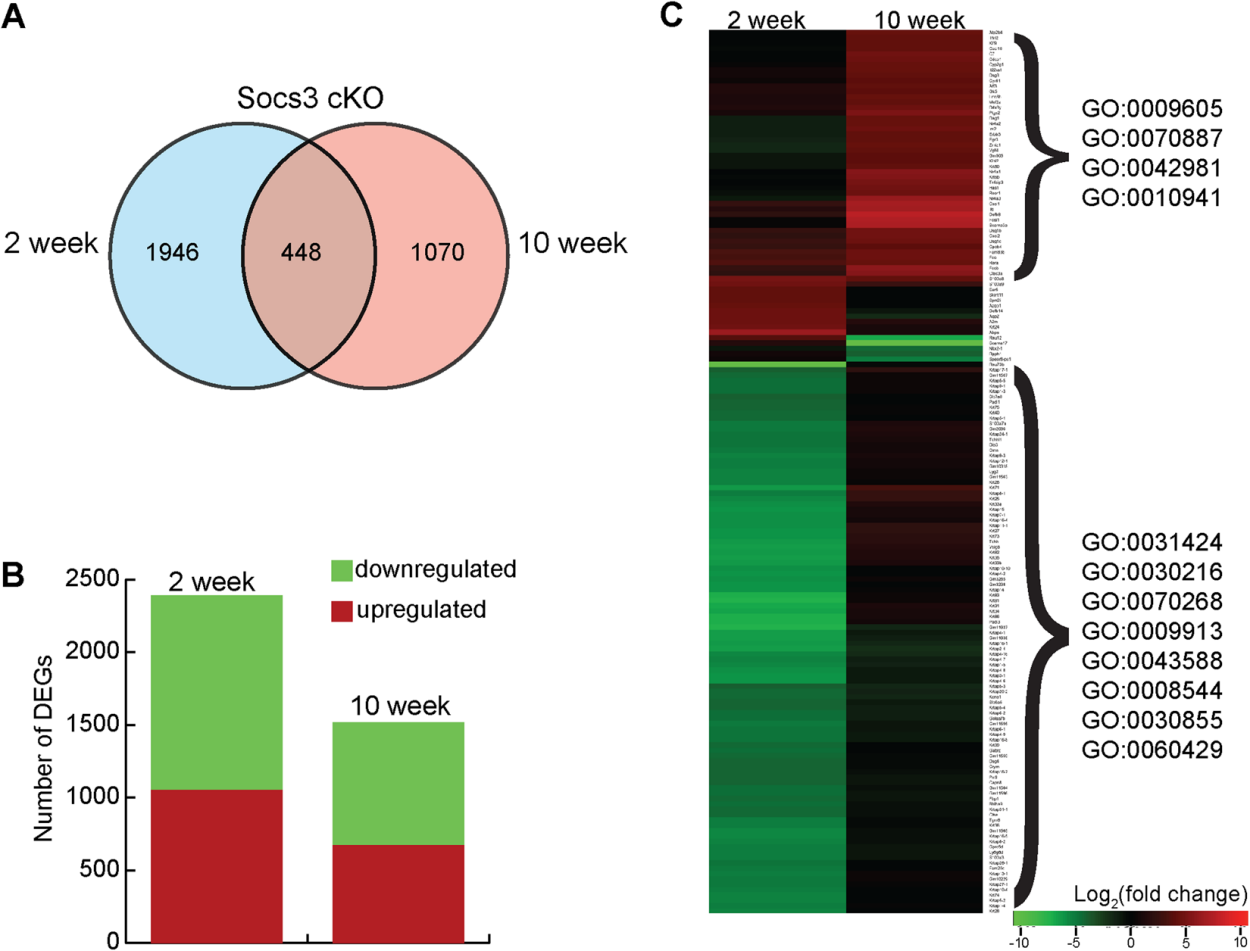
Gene expression profiles in *Socs3* cKO. **(A)** Venn diagrams indicating the numbers of differentially expressed genes (DEGs) between *Socs3* cKO and the wild type at two ages, 2 and 10 weeks [p < 0.05, FDR < 0.01, |log_2_(fold change)| ≥ −1]. **(B)** Bar chart representing the total numbers of genes that were up-regulated (red) or down-regulated (green) in *Socs3* cKO at both time points. **(C)** Heat map representing the expression profiles of the top 165 up-regulated (red) [FDR ≤ 0.01 and |log_2_(fold change)| ≥ 4] and down-regulated (green) (FDR  ≤ 0.01 and |log_2_(fold change)| ≥ 4) genes in 2- and 10-week-old *Socs3* cKO in comparison with the wild type. Wild type: 2 weeks, n = 6; 10 weeks, n = 4. *Socs3* cKO: 2 weeks, n = 4; 10 weeks, n = 4.

To investigate the global molecular perturbations associated with deletion of *Socs3* in keratinocytes, we compared gene expression between wild-type (C57BL/6 mice) and *Socs3* conditional knockout (*Socs3* cKO) mice at two different ages, 2 and 10 weeks. RNA-Seq reads were first aligned to the mouse genome (mm9) using TopHat2^17^. Raw gene counts were then normalized and expressed as frequency per kilobase per million mapped reads (FPKM) for 21110 genes annotated in the reference genome database^18,19^. Next, annotated genes were analyzed using the EdgeR package^20^ in the R statistical environment, and statistically significant differentially expressed genes (DEGs) were identified based on their calculated log_2_ fold changes [|log_2_(FC)| ≥ 1] and false discovery rates (FDR < 0.01) at both time points (Fig. 1A, Supplementary Table S1). A total of 2394 and 1518 genes were differentially expressed at 2 and 10 weeks, respectively (Fig. 1B). Approximately 50% of DEGs were either up-regulated or down-regulated at both 2 and 10 weeks (Fig. 1B), with 448 common DEGs (Fig. 1A). To highlight the major differences in the expression profiles of 2- and 10-week-old mice, we applied a stringent fold-change threshold [|log_2_(FC)| ≤ 4, FDR < 0.01] and generated a heat map of the 165 DEGs with the greatest perturbation in expression levels in either of the two samples (Fig. 1C). Clear distinctive patches of DEGs were found to be up- or down-regulated (Fig. 1C).

To further identify and characterize the molecular basis of DEGs in *Socs3* cKO samples, we performed Gene Ontology (GO) pathway enrichment analysis^21^. In the 2 week samples, the results revealed significant enrichment of skin-related GO terms, including keratinization (GO:0031424), keratinocyte differentiation (GO:0030216), cornification (GO:0070268), and epidermal cell differentiation (GO:0009913). By contrast, GO terms related to response to external stimulus (GO:0009605), cellular response to chemical stimulus (GO:0070887), regulation of apoptotic process (GO:0042981), and regulation of cell death (GO:0010941) were highly enriched in the 10 week samples.

### Dynamics of altered gene expression profiles in *Socs3* cKO keratinocytes at 2 and 10 weeks

**Figure 2:**
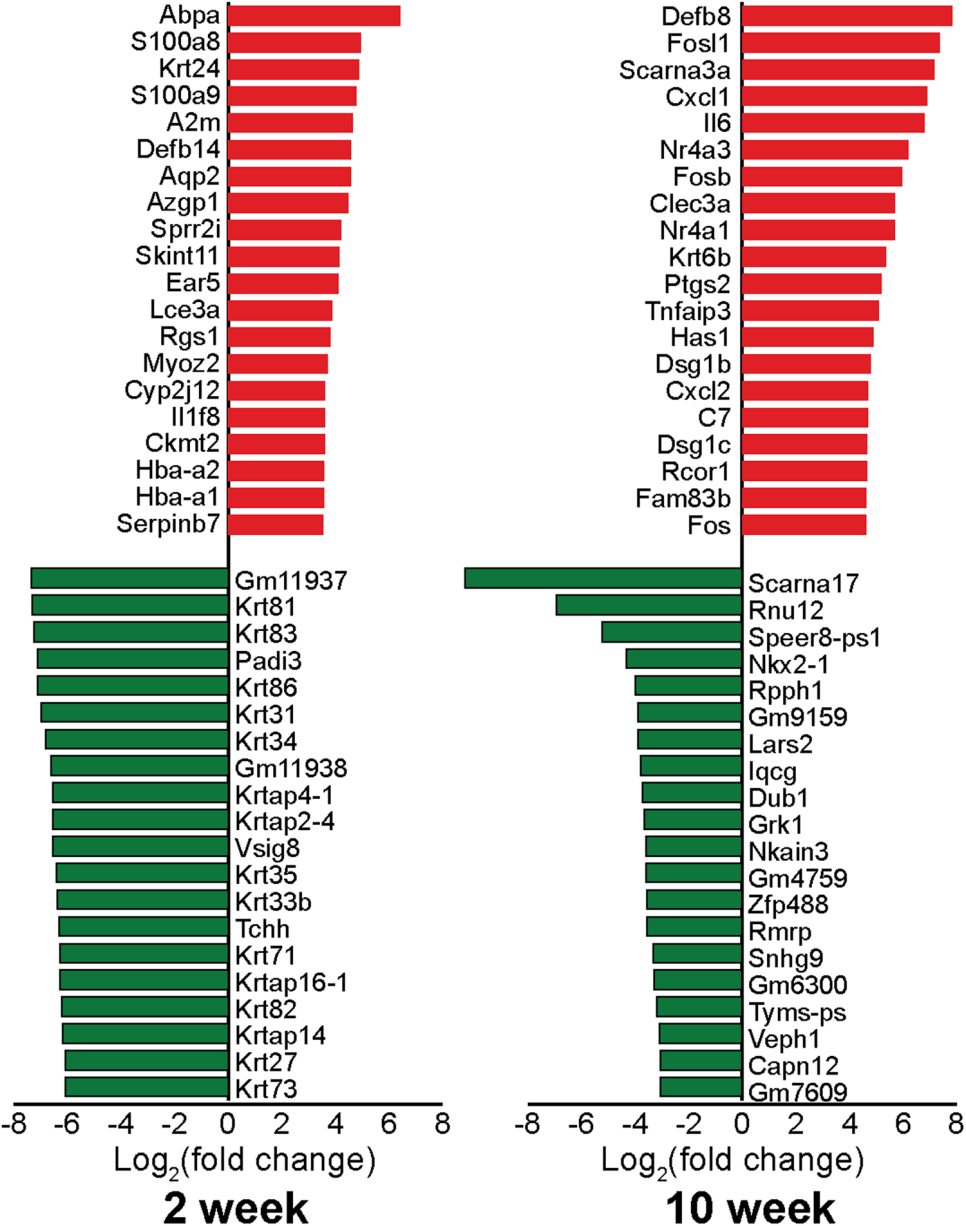
Differentially expressed genes (DEGs) in *Socs3* cKO samples. Top 20 up-regulated (red) and down-regulated (green) DEGs at 2 and 10 weeks in *Socs3* cKO [FDR ≤ 0.01 and |log_2_(fold change)| ≥ 2].

To further elucidate the role of *Socs3* in keratinocytes at early stages of development, we selected the top 20 most significant DEGs with the largest decreases and increases in transcript levels in 2- and 10-week-old *Socs3* cKO mice. As shown in Fig. 2 (left), all keratin-associated genes crucial for formation of the epidermis, keratinization, epidermal growth and barrier function, and formation of the cornified envelope were significantly down-regulated in 2-week-old *Socs3* cKO mice. *S100a8 and S100a9*, which encodes a calcium-binding protein also known as calprotectin, an early biomarker of psoriasis, were among the most up-regulated genes^22–25^. Also up-regulated were the genes encoding small proline-rich protein 2i (*Sprr2i*), serine protease (SP) inhibitor (*Serpinb7*), and beta defensin (*Defb14*), which play major roles in desquamation and control of skin barrier homeostasis^26–28^.

To understand the dynamics of gene expression in *Socs3* cKO mice, we then analyzed the data from the 10-week-old mice. In contrast with the younger mice, the most up-regulated genes in the older mice were enriched in functions related to acute immune responses, including multiple chemokines (*Cxcl1*, *Cxl2*), an interleukin cytokine (*Il-6*), *Fos*, *Fosl1*, and *Fosb* (Fig. 2, right). *Clec3a*, which encodes a C-type lectin family member that inhibits activation of CD4 + T cells and plays an important role in immune regulation, was up-regulated by 16-fold. The most down-regulated genes included *Dub1*(deubiquinating enzyme 1), *Nkx2-1*(homeodomain transcription factor; TF), *Zfp488*, and *Zfp457* (zinc finger proteins 488 457) (Fig. 2, right). Interestingly, *Defb14*, *Il-6*, and genes related to S100 are overexpressed in the skin disease initiated by loss of *Socs3* in keratinocytes, leading to a severe skin condition in older mice^13^. It is likely that these genes represent ‘first responders’ in which dramatic expression changes contribute to the onset of skin disease.

### Analysis of enriched function and disease-associated pathways in 2- and 10-week-old mice

**Figure 3:**
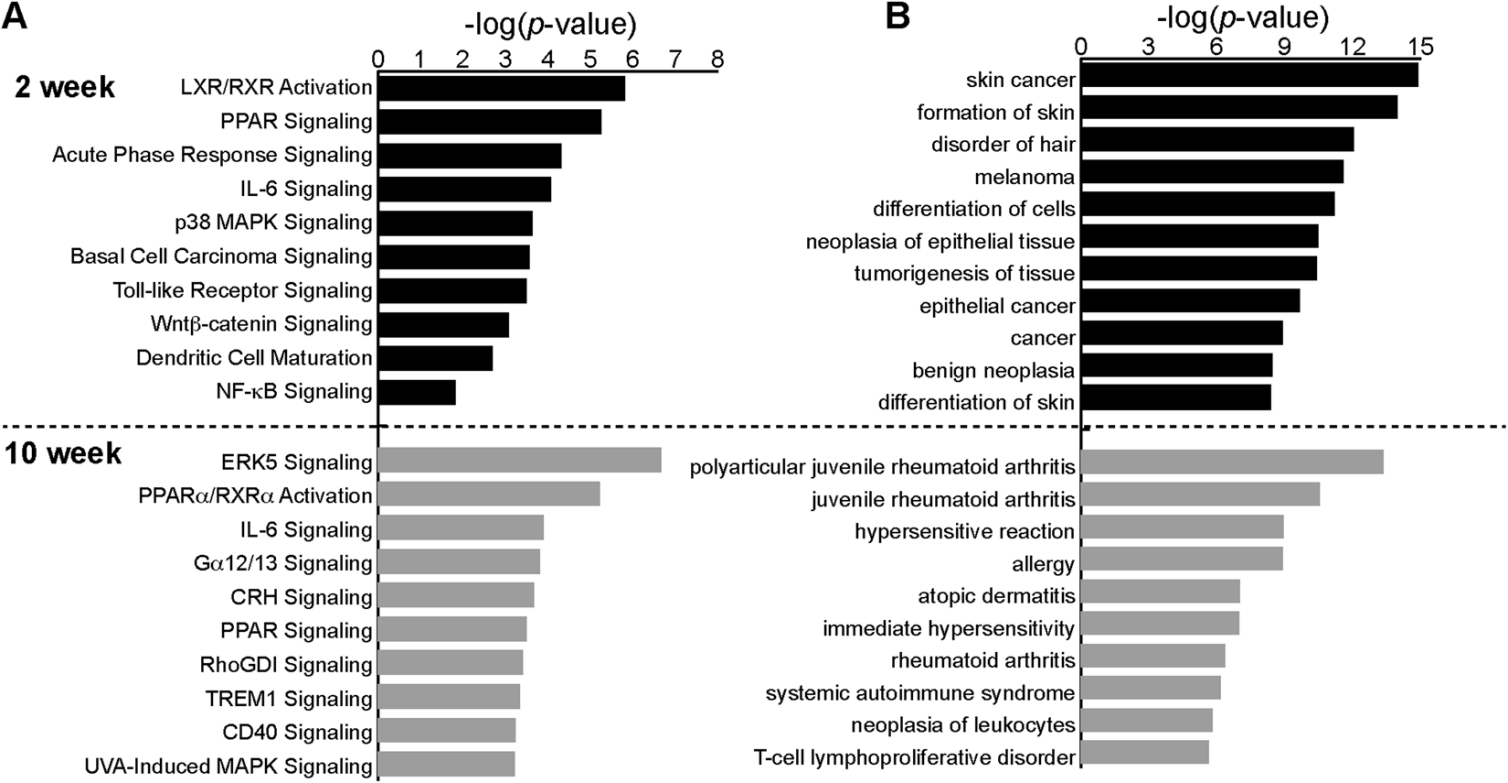
Enriched pathway and bio-function analysis of *Socs3* cKO. **(A)** Top 10 enriched canonical pathways, selected based on −log(p-value). **(B)** Functional annotation of the top enriched disease and bio-function analysis at 2 and 10 weeks. Bar size indicates the level of significance for each pathway [i.e., −log(p-value)].

Next, we used Ingenuity Pathway Analysis (IPA) to identify enriched biological processes and molecular functions altered by loss of *Socs3*. Comparison of wild-type and *Socs3* cKO DEGs at 2 weeks revealed that the most enriched pathways in the knockout animals were ‘LXR/RXR (liver X receptor/retinoid X receptor) signaling’ [−log(p-value) = 5.81] and ‘PPAR (peroxisome proliferator–activated receptors) signaling’ [−log(p-value) = 5.25] (Fig. 3A). The LXR/RXR and PPAR pathways regulate multiple genes involved in lipid biosynthesis and metabolism. In patients with inflammatory skin disorders or epidermal psoriasis, PPAR expression is significantly altered^29^. Dysregulation of lipid metabolism and oxidative pathways are among the early signatures of skin inflammation. In epidermal psoriasis, levels of total lipids, low-density lipoproteins (LDL), and phospholipids are significantly elevated, concomitant with activation of inflammatory responses mediated by the innate and adaptive immune systems^30–35^. Consistent with this, ‘acute phase response signaling’ [−log(p-value) = 4.32], ‘IL-6 (interleukin 6) signaling’ [−log(p-value) = 4.07], and ‘p38 MAPK signaling’ [−log(p-value) = 3.64] were also highly enriched at 2 weeks in *Socs3* cKO mice (Fig. 3A, Supplementary Table S2).

At 10 weeks, the top 10 enriched pathways included ‘ERK5 signaling’ [−log(p-value) = 6.66], ‘Gα12/13 signaling’ [−log(p-value) = 3.81], ‘RhoGDI Signaling’ [−log(p-value) = 3.41], ‘TREM-1 signaling’ (triggering receptor expressed on myeloid cells type-1) [−log(p-value) = 3.33], and ‘CD40 signaling’ [−log(p-value) = 3.23] (Fig. 3A, Supplementary Table S2). ERK-related family proteins play well-established roles as intracellular messengers during chronic inflammatory diseases, controlling multiple cellular processes that regulate cell differentiation, proliferation, and apoptosis, by binding the TFs AP-1 (activator protein-1) and nuclear factor-kappa B (NF-κB)^36–39^. TREM-1 signaling is dramatically induced in psoriasis and contributes to acute inflammation via recruitment of multiple cytokines^40^. CD40 signaling activates section of proinflammatory cytokines (TNF-a, IL-12), which promote T-cell survival and differentiation into the Th1 type in various autoimmune diseases^41,42^.

IPA Disease Bio-Function analyses pointed out the most significantly enriched diseases and biological functions at 2 weeks were associated with ‘skin cancer’ [194 target genes from DEGs; −log(p-value) = 14.88] and ‘formation of skin’ [37 target genes, −log(p-value) = 14] (Fig. 3B, Supplementary Table S3). By contrast, at 10 weeks the most enriched diseases and functions were ‘atopic dermatitis’ [22 target genes, −log(p-value) = 7], ‘hypersensitive reaction’ [34 target genes, −log(p-value) = 9], ‘T-cell lymphoproliferative disorder’ [30 target genes, −log(p-value) = 5.7], and ‘allergy’ [33 target genes, −log(p-value) = 8.9] (Supplementary Table S3). Taken together, these analyses suggest that the loss of *Socs3* affects formation and morphology of skin and initiates skin inflammation by activating various hypersensitivity reactions, followed by systematic autoimmune proinflammatory responses^11,43^.

### Expression of AP-1 family members is significantly altered in *Socs3* cKO keratinocytes

**Figure 4:**
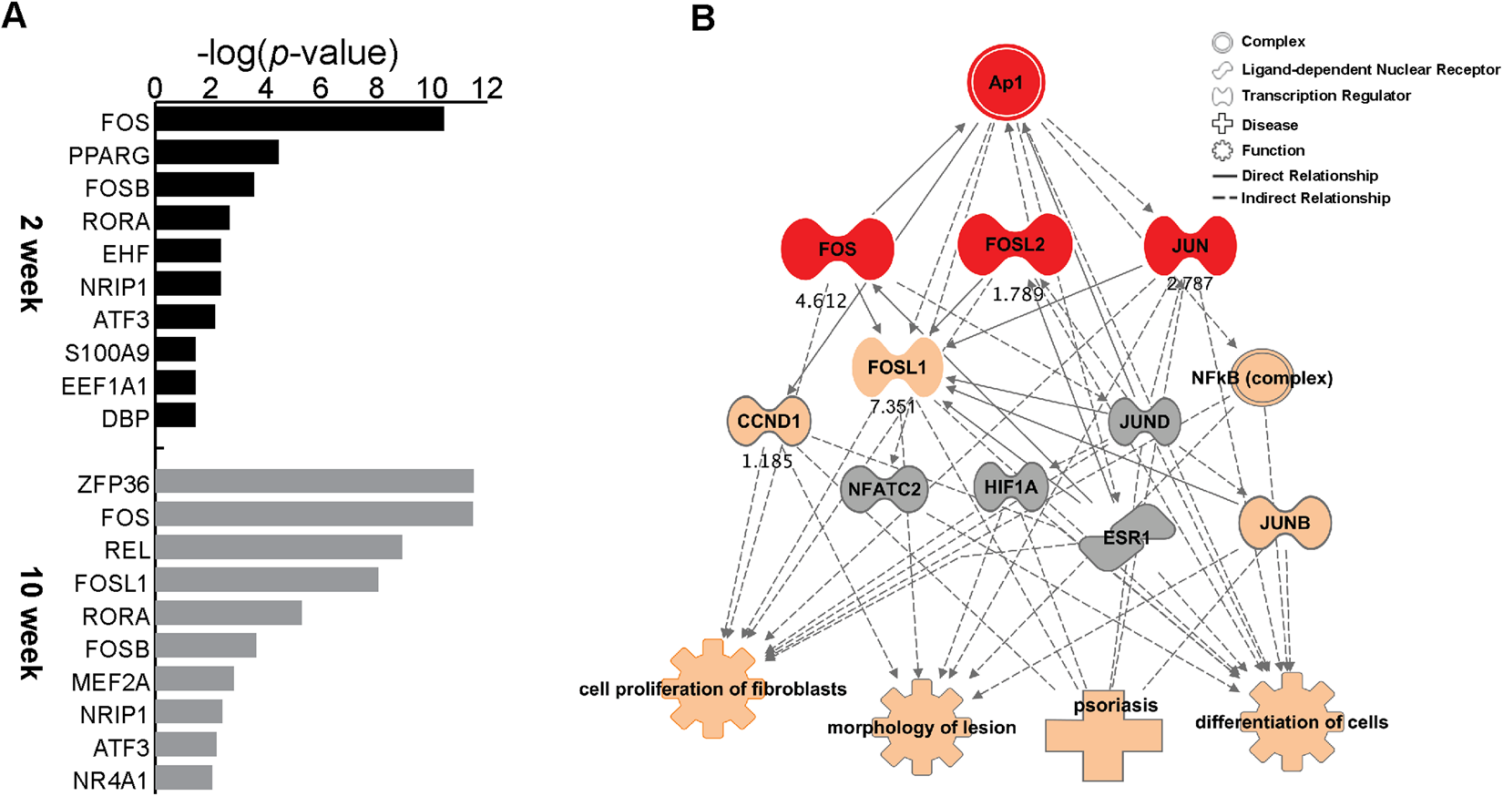
Transcription regulator network revealed that several regulators of AP-1 are altered in *Socs3* cKO. (**A)** Upstream analysis was performed using IPA software to identify highly regulated transcription factors at 2 and 10 weeks in *Socs3* cKO. Activator protein-1 (AP-1)-related transcription factors were differentially regulated at both time points. **(B)** Predicted mechanistic network of AP-1 regulation; numbers represent log_2_(fold change) of AP-1–related genes. Different structures of nodes represent different functional classes of gene products. Red indicates DEGs selected for validation. The nature of the relationship between nodes (direct or indirect) is indicated by a solid or dotted line, respectively.

Genes related to critical cellular functions are often under tight transcriptional control. To elucidate the core transcriptional machinery responsible for differential gene expression, we sought to identify potential TFs that were significantly enriched based on their fold change and overlap p-values (Supplementary Table S4) in 2- and 10-week-old *Socs3* KO mice. For the up-regulated DEGs, IPA revealed significant enrichment of targets involved in regulation of AP-1 signaling (*Fos*, *FosB*, *Atf3*, and *FosL1*) (Fig. 4A). AP-1 proteins play major roles in keratinocyte growth, proliferation, and apoptosis^44–49^. Moreover, various keratin family genes, along with pro-filaggrin and other epidermal growth factor–associated genes that are crucial for skin homeostasis, are also controlled by AP-1 family members^44,49–54^. Thus, perturbation of AP-1–mediated transcription leads to disrupted skin homeostasis and altered expression of cytokines and chemokines, eventually leading to disease phenotypes including hyperplasia, hyperkeratosis, psoriasis, and cancer^44,55,56^. Notably in this regard, we also identified PPARG, EHF (ETS subfamily homologue), and S100A9, an important regulator of genes involved in keratinocyte differentiation, lipid metabolism, and skin barrier homeostasis, among the top regulators in 2-week-old *Socs3* cKO mice, in accordance with the enriched canonical pathways shown in Fig. 3A.

To further elucidate the network of shared AP-1 TFs and their target molecules, we constructed an AP-1 TF regulatory network using the DEGs from 10-week-old mice. Figure 4B highlights some of the nearest neighbors of AP-1 TFs and the top associated biological functions and diseases. This analysis revealed that these TFs have important functions in dermatological diseases such as psoriasis (p-value = 4.29 × 10^−10^) and cellular processes associated with differentiation (p-value = 3.02 × 10^−12^), proliferation (p-value = 2.36 × 10^−13^), and lesion morphology (p-value = 1.14 × 10^−10^) (Fig. 4B). Collectively, our TF analyses demonstrate the importance of AP-1–related TFs in *Socs3* cKO, and identified several AP-1 TFs (*fos*, *fosl2*, *Jun*) worthy of validation in human cell culture.

### *SOCS3* depletion causes time-dependent up-regulation of mRNAs encoding AP-1 elements in cultured human keratinocytes

**Figure 5:**
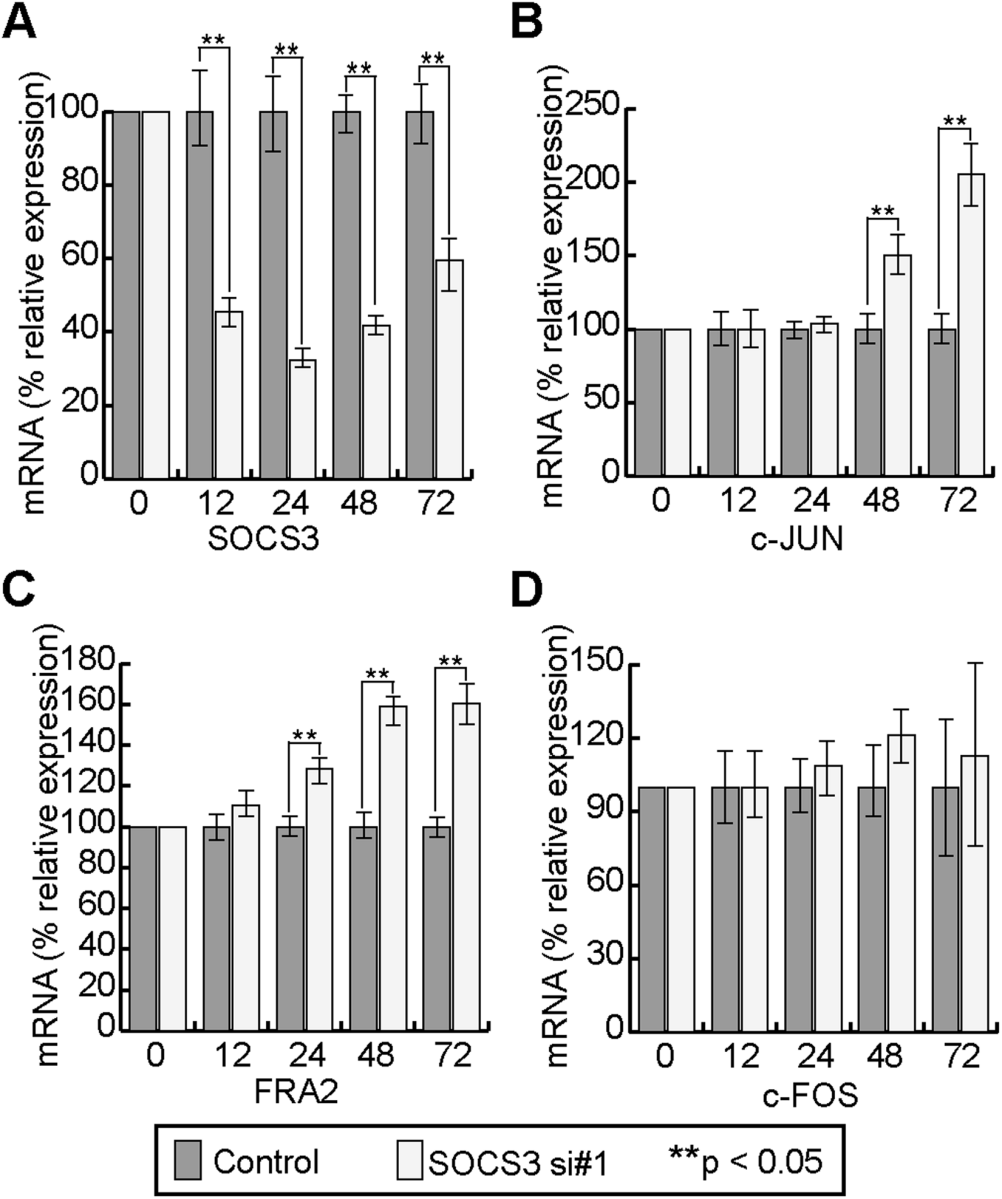
Differential expression of AP-1 genes upon SOCS3 depletion. **(A–D)** HaCaT cells were transfected with SOCS3 or control siRNA. At 0 (pre-transfection), 12, 24, 48, and 72 h post-transfection, mRNA levels of *SOCS3* **(A)**, *JUN* **(B)**, *FRA2* **(C)**, and *FOS* **(D)** were quantitated by qRT-PCR. mRNA levels were normalized against β-actin mRNA. (n = 5, p < 0.05).

Our RNA-Seq analyses suggested the involvement of *Socs*3 in regulation of AP-1 TF genes. To extend these findings, we asked whether this regulatory connection could also be observed in *SOCS3*-knockdown human keratinocytes. To this end, we used two different siRNAs specifically targeting *SOCS3* mRNA (SOCS3 si#1 and SOCS3 si#2) to decrease expression of *SOCS3* in HaCaT cells, and then used quantitative real-time PCR to measure the mRNA levels of AP-1–related TFs at various time points after transfection. Transfection with SOCS3 si#1 decreased *SOCS3* mRNA expression to 45.6%, 32.4%, 41.8%, and 59.5% of the control level at 12, 24, 48, and 72 h post-transfection, respectively (Fig. 5A). The SOCS3 protein level was also reduced at later time points (67.9% and 49.0% of the control level at 48 and 72 h post-transfection, respectively, but no significant reduction at 12 or 24 h) (Supplementary Figure S1).

We then investigated the effect of *SOCS3* depletion on mRNA levels of genes encoding AP-1 factors such as c-JUN (*JUN*), c-FOS (*FOS*), and FRA2 (*FOSL2*). All of these genes were affected by *Socs3* depletion in different manners and to varying extents, suggesting that *Socs3* indeed regulates AP-1 genes (Fig. 5). The mRNA levels of *c-JUN* and *FRA*2 increased in a time-dependent manner following *SOCS3* depletion (Fig. 5B,C). The *FRA2* mRNA level in SOCS3 siRNA–transfected cells was similar to that in control cells 12 h post-transfection, but then started to rise over time (28.5%, 58.8%, and 60.2% higher than the control level at 24, 48, and 72 h post-transfection, respectively) (Fig. 5C). The *JUN* mRNA level exhibited a remarkable time-dependent increase under *SOCS3* depletion, reaching levels 50.2% and 105.3% higher than those in control cells at 48 and 72 h post-transfection, respectively, although this increase was not apparent at 24 h post-transfection (Fig. 5B). By contrast, the *FOS* mRNA level was slightly elevated at 42 and 72 h post-transfection, but the effect was not significant (Fig. 5D). Taken together, these observations suggest a difference in the regulation of AP-1 subunits genes by SOCS3, possibly mediated by SOCS3-dependent regulatory pathways as reported previously in keratinocytes^44,57,58^. Similar mRNA patterns for *JUN*, *FRA2*, and *FOS* were observed in cells transfected with SOCS3 si#2 (Supplementary Figure S2).

To confirm that the increase in AP-1 gene expression was reflected by a corresponding increase in protein expression, we measured the c-JUN protein level in SOCS3-knockdown (using SOCS3 si#1) HaCaT cells at 72 h post-transfection (Supplementary Figure S3). SOCS3-knockdown HaCaT cells exhibited significant increases in both phosphorylated and un-phosphorylated c-JUN protein, further supporting our RNA-Seq and qRT-PCR observations. Given that SOCS3 si#1 decreased the SOCS3 protein level only after 24 h, the *JUN* and *FRA2* mRNA levels seemed to most closely reflect the change in SOCS3 protein level among the AP-1 genes we tested, suggesting a link between *SOCS3* and AP-1–related TFs such as c-JUN.

## Discussion

To obtain a basic understanding of the role of *Socs3* in epidermal homeostasis, we generated and analyzed transcriptome profiles from mice in which *Socs3* was conditionally knocked out in keratinocytes. RNA-Seq data from *Socs3* cKO mice at 2 and 10 weeks of age were analyzed to identify the most significantly dysregulated genes and their associated molecular pathways. The results revealed that a series of molecular perturbations in the epidermis began at 2 weeks that ultimately led to a skin disease–like phenotype^13^, indicating that *Socs3* plays an important role in skin barrier homeostasis. Loss of *Socs3* in keratinocytes resulted in down-regulation of several genes related to epidermal development and barrier function at a very early age (2 weeks), followed by perturbation in genes associated with activation of multiple proinflammatory cytokines and stress-related pathways at 10 weeks (Figs 1 and 2). AP-1 TFs, in particular c-Jun, were highly up-regulated in later life in cKO mice, and this up-regulation was verified in SOCS3-knockdown HaCaT human primary keratinocytes (Figs 4 and 5).

In the epidermis, keratinocytes perform multiple complex tasks, including coordinated proliferation, differentiation, and orchestrated immune responses to intercommunicate with other cell types, all of which contribute to maintenance of skin homeostasis^59,60^. Our RNA-Seq analyses demonstrated that the expression levels of various genes involved in these highly specialized molecular events were significantly affected in *Socs3* cKO mice (Figs 1 and 2) at an early stage of development (2 weeks). At that time point, most keratin genes were drastically down-regulated, and the expression of genes involved in maintenance of skin barrier function (e.g., SPs, *Sprr2i*, *Serpinb7*) was altered (Fig. 2). The impact of impaired epidermal organization is likely to be exacerbated by hyper-activation of immune and stress responses (Fig. 2; *S100A8*, *S100A9*, beta defensin, *IL-6*), reflecting invasion by microbes or irritants^61–65^, and up-regulation of lipid metabolism, reflecting the high demand for lipid synthesis to compensate for reduced barrier hydrophobicity^66^ (Fig. 2). Thus, the epidermis had reached a pre-disease state in young *Socs3* KO mice.

Numerous studies using animal models have demonstrated the critical importance of *Socs3* in restraining inflammatory skin diseases^13,14,67,68^. Under normal conditions, *Socs3* works as an anti-inflammatory checkpoint that controls the regulation of various cytokine-triggered immune responses by inhibiting IL-6–induced activation of the STAT3 signaling pathway^4,13,14,67,68^. However, hyper-activation of STAT3 in keratinocytes under SOCS3 depletion induces higher levels of proinflammatory cytokines IL-19, IL-20, and IL-24, resulting in severe inflammation, hyperplasia, and keratinocyte hyper-proliferation^13^. Moreover, double knockout of *Socs3* and *Il-6*, but not *Il-4* and *Il-13*, rescues skin lesions induced by *Socs3* KO^13^. In addition, Socs3 negatively regulates uncontrolled interferon-γ (IFN-γ) signaling, which is responsible for severe inflammation in various cell types^69–74^. Taken together, these findings suggest that Socs3 prevents disruption of homeostasis, which would impair skin barrier function. Thus, the ‘snapshots’ of disease progression extracted from our findings suggest a sequence of latent, but highly dynamic, molecular shifts in *Socs3* cKO keratinocytes, starting from an early stage and ultimately leading to disease phenotype at later time points. This suggests in turn that *Socs3* deficiency triggers hyper-activation of immune responses, leading to impaired skin barrier homeostasis at 15 weeks^13^. Although our findings elucidate some of the key phenomena that occur during the early stage of skin disease progression, further study will be required to elucidate the detailed mechanism.

AP-1 TFs play important roles in differentiation and proliferation of epidermal keratinocytes^44,45,47,53,54,75,76^. Consistent with this, disruption of AP-1 function affects the onset of various diseases phenotypes, including psoriasis development and atopic dermatitis. The link between Socs3 and AP-1 transcriptional activity has been reported previously^77^. Socs3 suppresses AP-1 activity by inhibiting c-JUN phosphorylation in neuroblastoma cells^77^. On the other hand, Socs3 depletion increases AP-1–related transcript levels in oligodendrocytes^78^. Moreover, elevated expression of *c-Jun* in the suprabasal epidermis results in extensive hyperplasia and hyperkeratosis, which are hallmarks of psoriatic skin^79–81^. Our transcriptome analysis suggests that *Socs3*-dependent AP-1 transcriptional regulation might play a significant role in maintaining epidermal homeostasis, although the detailed regulatory mechanisms remain to be elucidated. It is possible that the induction of AP-1–related genes is due to early activation of p38 MAPK signaling (Fig. 3A), which is involved in the regulation of both gene groups in various contexts^82–86^.

Taken together, our results reveal novel regulatory dynamics of *Socs3*, which plays a pivotal role in regulating important genes involved in skin homeostasis. Our observations provide insights into the mechanisms underlying maintenance of skin homeostasis, in which *Socs3* (in cooperation with other genes) orchestrates multiple essential events such as keratinocyte proliferation, differentiation, and immune or stress responses. In addition, experimental validation of our computation-driven hypothesis in human keratinocytes confirmed the role of *Socs3* in the regulation of AP-1 TF genes. Future studies, including detailed time-series analysis and modeling of molecular interactions, will help us to understand the role of *Socs3* dynamics in the maintenance of skin homeostasis and skin disease progression.

## Materials and Methods

### Animal model

The keratin 5–specific *Socs3* cKO mouse was generated in the C57BL/6 background as described previously^13^. All mice used in this study were maintained in specific pathogen–free (SPF) conditions. Prior approval for animal experiments was obtained from the Animal Research Ethics Committee of RIKEN Yokohama, Japan. All mice were maintained and studied in accordance with the guidelines of the RIKEN Animal Research Committee.

### RNA-Seq sample preparation

Total RNA was isolated using TRIzol (Thermo Fisher Scientific, Waltham, MA, USA) from ear samples of wild-type (n = 6 at 2 weeks, and n = 4 at 10 weeks) and *Socs3*-deficient mice (n = 4 at 2 weeks, and n = 4 at 10 weeks) at 2 or 10 weeks of age. cDNA libraries were synthesized using the TruSeq RNA Library Preparation Kit v2 (Illumina, San Diego, CA, USA). Sequencing data were generated on a HiSeq. 1000 system (Illumina) as single-ended 50-base reads. EdgeR package^20^ in the R statistical environment was used to analyze DEGs. The Orange software^75^ was used to draw Venn diagrams to depict DEGs at both time points.

### Data availability statement

All data related to this study have been deposited in the public repository Gene Expression Omnibus (GEO) (http://www.ncbi.nlm.nih.gov/geo/) with accession number GSE94743.

### Cell culture and antibodies

The human keratinocyte–derived HaCaT cell line was maintained at 37 °C with 5.0% CO_2_ in Dulbecco’s modified Eagle’s medium (DMEM) with high glucose and high pyruvate (Gibco) supplemented with 10% fetal bovine serum (Gibco), 100 U/ml penicillin (Gibco), and 100 U/ml streptomycin (Gibco). Rabbit anti-SOCS antibody (#2923, Cell Signaling Technology), rabbit c-Jun (60A8) antibody (#9165, Cell Signaling Technology), rabbit Phospho-c-Jun (Ser63) (54B3) antibody (#2361, Cell Signaling Technology), and mouse anti-β-actin antibody [Anti-BETA-Actin (C4): sc-47778; Santa Cruz Biotechnology] were used for immunoblot analysis. Horseradish peroxidase (HRP)-conjugated sheep anti-mouse IgG and donkey anti-rabbit IgG were purchased from GE Healthcare.

### Socs3 knockdown in HaCaT cells

Two different siRNAs targeting *SOCS3* mRNA were used to knock down SOCS3 in HaCaT cells. *SOCS3* si#1 (5′-CGCUCAGCGUCAAGACCCAdTdT-3′ and 5′-UGGGUCUUGACGCUGAGCGdTdT-3′) and a control siRNA (5′-CCUACGCCACCAAUUUCGUdTdT-3′ and 5′-ACGAAAUUGGUGGCGUAGGdTdT-3′) were synthesized by Eurofins Genomics. The second siRNA (*SOCS3* si#2) against target sequence ‘CACCUGGACUCCUAUGAGA’ was purchased from ON-TARGETplus Human SOCS3 (9021) siRNA (GE Dharmacon^™^). Both siRNAs were transfected into cells using INTERFERin (Polyplus-transfection, New York, NY, USA). SOCS3 knockdown was confirmed by measurement of *SOCS3* mRNA (for both *Socs3* si#1 and si#2) and SOCS3 protein levels (for Socs3 si#1 only) at 12, 24, 48, and 72 h post-transfection. *SOCS3* si#1, n = 5; *SOCS3* si#2, n = 3.

### Quantitative real-time PCR (qRT-PCR)

Total RNA was prepared from cells on a Maxwell 16 Automated Purification System (Promega) using the Maxwell LEV simplyRNA Tissue Kit (Promega), and then subjected to reverse transcription to synthesize complementary DNA (cDNA) using ReverTra Ace qPCR RT Master Mix (TOYOBO). Specific primers for the SOCS3, β-actin, and AP-1 genes were synthesized by Eurofins Genomics (Supplementary Figure S4). cDNA was amplified on a LightCycler 480 II instrument (Roche) with LightCycler 480 SYBR Green I Master (Roche) under the following reaction conditions: 45 cycles of 95 °C for 10 sec, 60 °C for 10 sec, and 72 °C for 10 sec. Relative mRNA levels were determined based on the ∆∆Ct method, and normalized against β-actin mRNA

### Enriched functional pathway and transcriptional regulator analysis

Canonical biological pathways were identified using the IPA software (www.qiagen.com/ingenuity). Fisher’s exact test method (fold change ≥ 2) was used to determine the probability of each disease and biological pathway based on genes differentially expressed at 2 and 10 weeks. For functional network and upstream transcriptional analysis, DEGs (fold change ≥ 2) were clustered into networks of known connections, biological functions, and associated diseases (p-values ≤ 0.05), based on a knowledge database and published references. The analysis identified how many known targets of the TFs were present in the *Socs3* cKO dataset, and also the degree of change with respect to the WT. Overlap p-value was computed based on significant overlap between DEGs in the dataset and the known targets of transcriptional regulators.

## Electronic supplementary material

Supplementary Figure

Supplementary Table S1

Supplementary Table S2

Supplementary Table S3

Supplementary Table S4
