# Supplementary material for: Insights into gene expression profiles induced by Socs3 depletion in keratinocytes
Source: Sci Rep. 2017 Nov 20;7:15830. doi: 10.1038/s41598-017-16155-1 (PMC5696538; doi:10.1038/s41598-017-16155-1)
Supplement: Supplementary file 1 — Supplementary Figure [file 41598_2017_16155_MOESM1_ESM.pdf]

Title: Insights Into Gene Expression Profiles Induced By *Socs3* Depletion In Keratinocytes

Archana Bajpai*[1], Takashi Ishii[1], Kosuke Miyauchi[2], Vipul Gupta[1,3], Yuka Nishio-Masaike[1], Yuki Shimizu-Yoshida[1,5], Masato Kubo[2,4], Hiroaki Kitano*[1,3,5,6]

[1]RIKEN-IMS, Laboratory for Disease Systems Modeling, Japan. [2]RIKEN-IMS, Laboratory for Cytokine Regulation, Japan. [3]The Systems Biology Institute, Japan. [4]Division of Molecular Pathology, Research Institute for Biomedical Science, Tokyo University of Science, Japan. [5]Sony Computer Science Laboratories, Inc, Japan. [6]Okinawa Institute of Science and Technology, Japan.

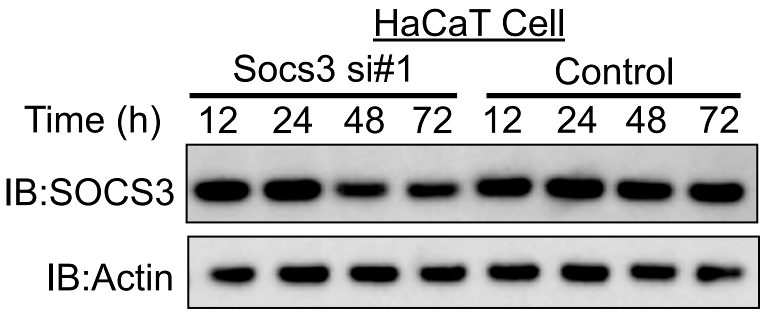

Supplementary Figure S1: HaCaT cells transfected with either Socs3 siRNA (Socs3 si#1) or a control siRNA (Control). Cells were subjected to immunoblot analysis after 12, 24, 48 and 72 h post transfection.

Title: Insights Into Gene Expression Profiles Induced By *Socs3* Depletion In Keratinocytes

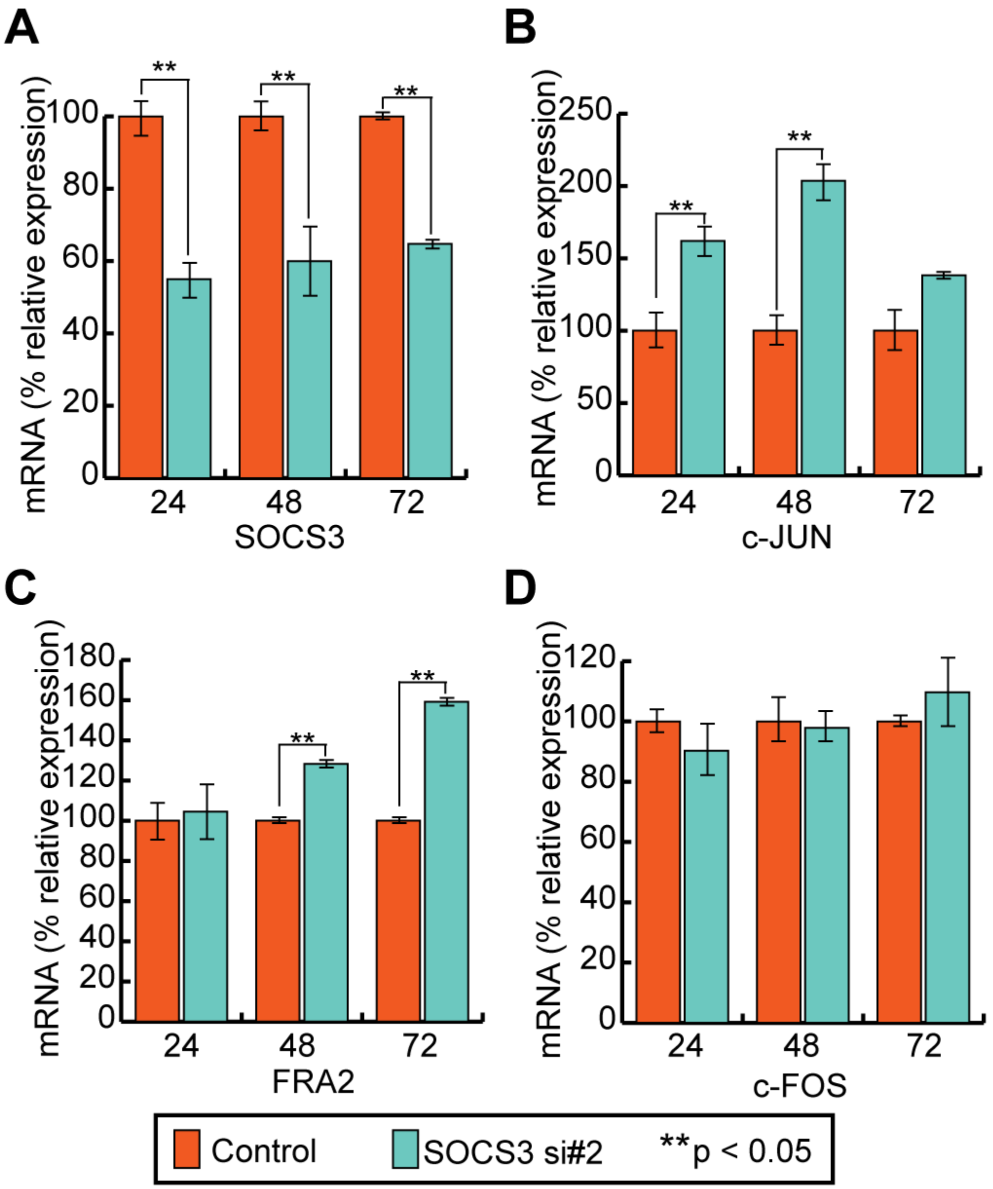

Supplementary Figure S2: Differential expression of AP-1 genes upon SOCS3 depletion: (A–D) HaCaT cells were transfected with SOCS3 si#2 or control siRNA. At 24, 48, and 72 h post-transfection, mRNA levels of SOCS3 (A), *c-Jun* (B), *Fra2* (C), and *c-Fos* (D) were quantitated by qRT-PCR. Experiment was performed in triplicates (n=3), and mRNA levels in each transcripts were normalized against β-actin.

Title: Insights Into Gene Expression Profiles Induced By *Socs3* Depletion In Keratinocytes

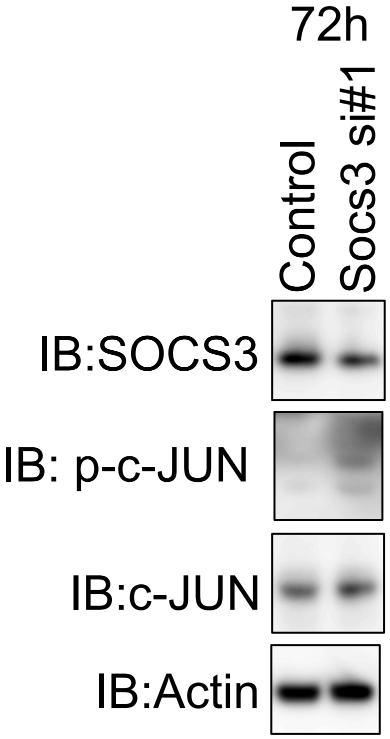

Supplementary Figure S3: HaCaT cells transfected with either Socs3 siRNA (Socs3 si#1) or a control siRNA (Conrol). Cells were subjected to immunoblot analysis after 72 h post transfection.

Gene	Forward	Reverse
SOCS3	GACCAGCGCCACTTCTTCA	CTGGATGCGCAGGTTCTTG
ß-actin	AGAGCTACGAGCTGCCTGAC	AGCACTGTGTTGGCGTACAG
c-Jun	GAAGTGTCCGAGAACTAAAG	AAAAGTCCAACGTTCCGTTC
c-Fos	ATGTTCTCGGGCTTCAACGCA	ATGAAGTTGGCACTGGAGACGG
Fra2	AACCCATACCCTCGCTCGCA	GCTGCAGCCAGCTTGTTCCT

Supplementary Figure S4: Table of forward and reverser primers used in this study.

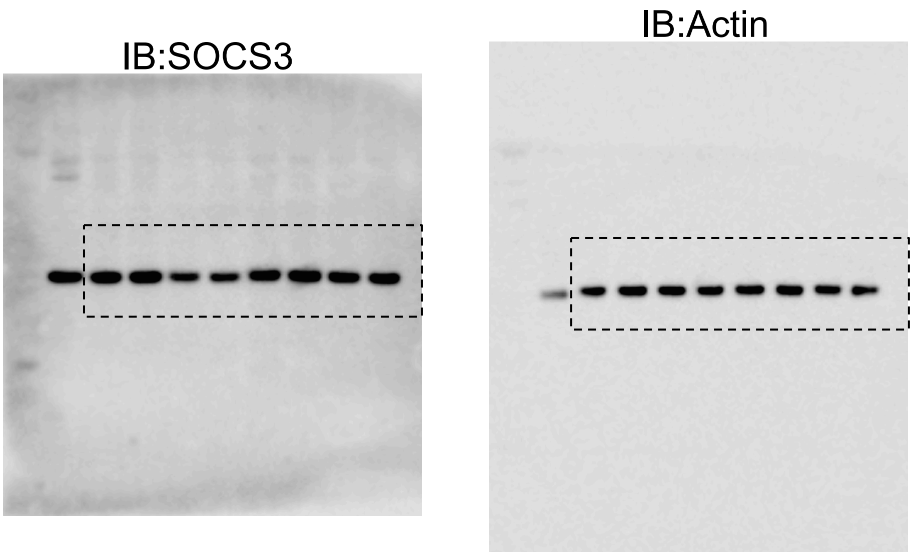

Supplementary Figure S5: Original immunoblot data for Supplementary Figure S1. Broken line rectangle borders the used image data.

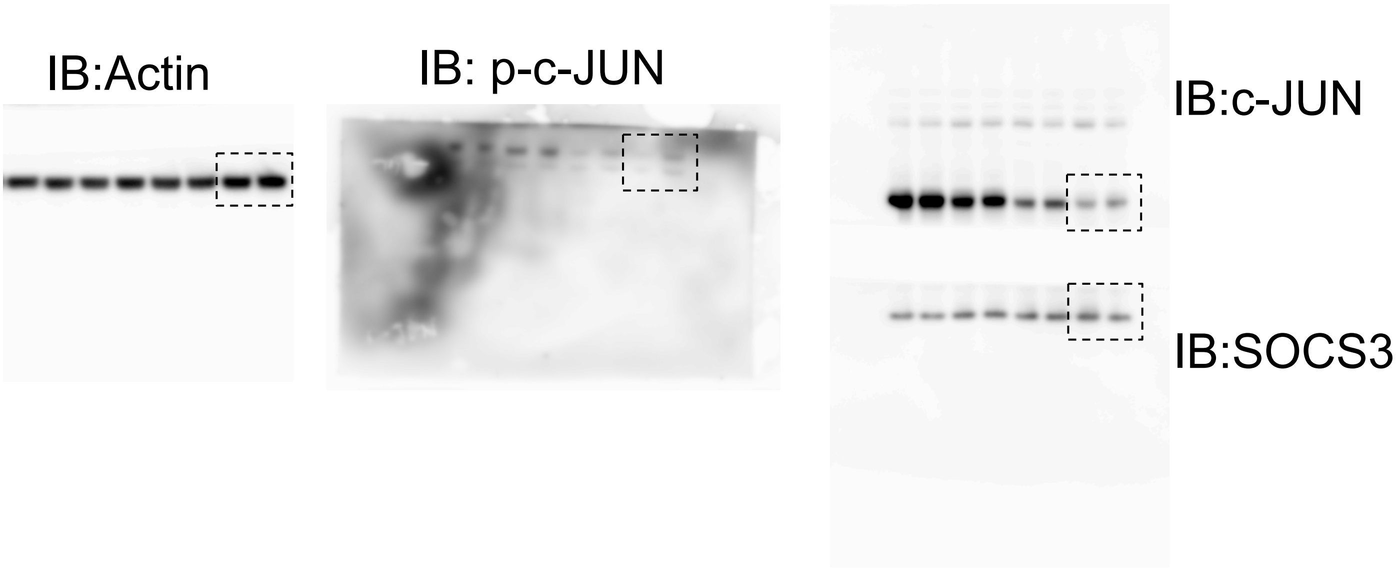

Supplementary Figure S6: Original immunoblot data for Supplementary Figure S3. Broken line rectangle borders the used image data.
